# Haemolytic Uraemic Syndrome Triggered by Non-Shiga Toxin-Producing Enteropathogenic Escherichia coli in a Child: Difficulties in Diagnosis and Treatment

**DOI:** 10.7759/cureus.104439

**Published:** 2026-02-28

**Authors:** Margarida Caldeira, Fiona Caldeira, Inês Pereira Soares, Rute Baeta Baptista, Telma Francisco

**Affiliations:** 1 Pediatrics Department, Hospital de Santarém, Unidade Local de Saúde da Lezíria, Santarém, PRT; 2 Pediatrics Department, Hospital Central do Funchal, Funchal, PRT; 3 Pediatrics Department, Hospital de Vila Franca de Xira, Unidade Local de Saúde do Estuário do Tejo, Vila Franca de Xira, PRT; 4 Pediatric Nephrology Department, Hospital Dona Estefânia, Unidade Local de Saúde de São José, Lisboa, PRT

**Keywords:** acute kidney injury, eculizumab, enteropathogenic escherichia coli, haemolytic uraemic syndrome, neurological involvement

## Abstract

Haemolytic uraemic syndrome (HUS) is an important cause of acute kidney injury (AKI) in children, most commonly triggered by Shiga toxin-producing *Escherichia coli* (STEC). In contrast, HUS associated with non-Shiga toxin-producing enteropathogenic *E. coli* (EPEC) is rare, particularly when accompanied by neurological involvement. Optimal management in such cases remains uncertain, especially concerning the role of complement inhibition.

A previously healthy three-year-old boy presented with bloody diarrhoea, abdominal pain, and fever. He developed biochemical features of HUS and neurological manifestations, including haemiparesis and seizures. Microbiological testing revealed an EPEC strain positive for the *eae* gene but negative for Shiga toxin. Despite the absence of complement gene pathogenic variants, treatment with plasma exchange (PLEX) and eculizumab (ECZ) was initiated due to rapid clinical deterioration. The patient achieved complete recovery of renal, haematological, and neurological function. Genetic analysis identified variants of uncertain significance, and ECZ discontinuation is being cautiously approached with extended dosing intervals.

Although ECZ is well established in complement-mediated HUS, its use in infection-associated HUS is controversial due to limited high-quality trial data. Nevertheless, emerging evidence and case reports suggest that complement activation may play a role in the pathogenesis of unusual forms. This case highlights the potential benefit of early complement inhibition in severe EPEC-associated HUS with neurological complications.

This case expands the spectrum of infectious triggers associated with HUS and supports a potential role for complement inhibition in selected cases of infection-associated disease, even without proven complement dysregulation. A personalised approach is vital in managing complex presentations. Further research is required to clarify treatment strategies and identify biomarkers predictive of therapeutic response.

## Introduction

Haemolytic uraemic syndrome (HUS) is a form of thrombotic microangiopathy (TMA) characterised by non-autoimmune microangiopathic haemolytic anaemia, thrombocytopenia, and acute kidney injury (AKI). It represents one of the leading causes of paediatric renal failure [1].

Although the kidneys are primarily affected, HUS may also cause ischaemic injury in other organs, including the brain, gastrointestinal tract, and heart, among others. Consequently, clinical manifestations vary according to the organs involved [2]. Neurological symptoms are the most frequent extra-renal manifestations and are associated with increased morbidity and mortality [3].

HUS can be classified into several categories, the most common being infection-associated HUS (IA-HUS), typically caused by Shiga toxin-producing *Escherichia coli *(STEC), although other infectious agents, such as *Streptococcus pneumoniae*, have also been implicated. In contrast, complement-mediated HUS (CM-HUS) arises from the dysregulation of the alternative complement pathway, which may result from pathogenic variants in complement regulatory genes or the presence of autoantibodies directed against complement factors. Other less frequent causes include malignancy, medication-related HUS, and hereditary forms, such as inborn errors of cobalamin C (CblC) metabolism or pathogenic variants in the diacylglycerol kinase epsilon (DGKE) gene [4].

Regarding treatment, beyond supportive care, specific therapy depends on the underlying aetiology. In CM-HUS, management has been revolutionised by the introduction of eculizumab (ECZ), a humanised monoclonal IgG antibody that binds to the complement protein C5, thereby inhibiting its cleavage and preventing the formation of the terminal complement complex. This mechanism attenuates endothelial injury and microvascular thrombosis. ECZ has been shown to achieve haematological normalisation and preserve renal function, and it is now considered the standard of care for CM-HUS, having largely replaced plasma exchange (PLEX) in most cases [5,6].

Nevertheless, the role of ECZ in IA-HUS remains controversial, with conflicting results reported across several studies and no systematic reviews confirming a clear benefit. However, an increasing number of case reports have described favourable outcomes following ECZ use in children with neurological involvement secondary to STEC-HUS [5,7].

Although STEC-HUS is the main cause of IA-HUS, these authors found no cases in paediatric patients triggered by non-Shiga toxin-producing *E. coli* strains. We present the case of a child with HUS associated with a non-Shiga producer enteropathogenic *E. coli *(EPEC) who developed significant neurological complications and responded favourably to ECZ therapy.

This article was previously presented as an oral communication at the Portuguese Pediatric Nephrology Meeting in September 2025.

## Case presentation

A previously healthy three-year-old boy presented to the emergency department with a four-day history of abdominal pain, bloody diarrhoea, anorexia, nausea, and fever. On physical examination, he was dehydrated, lethargic, hypertensive (blood pressure: 111/75 mmHg), and anuric. There was no relevant family history for the present illness.** **Vaccinations were updated according to the Portuguese National Vaccination Programme, supplemented with vaccines against meningococcal ACWY and rotavirus vaccines.

Laboratory findings at admission revealed anaemia with schistocytes, thrombocytopenia, and AKI (according to the Kidney Disease: Improving Global Outcomes (KDIGO); baseline creatinine of 0.5 mg/dL; estimated glomerular filtration rate according to Schwartz's formula of 26.11 mL/min/1.73 m^2^). Other findings included leukocytosis with neutrophilia, elevated lactate dehydrogenase, hyperuricaemia, hyponatraemia, raised C-reactive protein, and metabolic acidosis on blood gas analysis (Table 1).

**Table 1:** Admission results RBC: red blood cells; HB: haemoglobin; HTC: haematocrit; MCV: mean corpuscular volume; MCH: mean corpuscular haemoglobin; MCHC: mean corpuscular haemoglobin concentration; RDW: red cell distribution width; WBC: white blood cells; Na: sodium; K: potassium; Cl: chloride; LDH: lactate dehydrogenase; CRP: C-reactive protein; PCO2: partial pressure of carbon dioxide; PO2: partial pressure of oxygen; HCO3: bicarbonate; BE: base excess; Ca: calcium; Glu: glucose; Lac: lactate; N/A: not applicable

Test	Result	Normal range	Unit
Laboratory evaluation			
RBC	3.53	3.80-5.40	10^6^/µL
HB	8.4	11-14	g/dL
HTC	24.8	32-42	%
MCV	70.3	72-86	fL
MCH	23.8	25-31	pg
MCHC	33.9	31.5-36	g/dL
RDW	15.5	11.2-13.4	%
WBC	26.25	4.5-17	10^3^/µL
Neutrophils	70.1	40.1-67.7	%
Lymphocytes	24.1	23.6-48	%
Monocytes	5.6	4.8-10.2	%
Eosinophils	0	0.8-5.5	%
Basophils	0.2	0.4-1.4	%
Platelets	21	173-360	10^3^/µL
Haptoglobin	<0.10	0.3-2	g/L
Creatinine	1.98	0.7-1.3	mg/dL
Urea	145	19-49	mg/dL
Uric acid	15.5	1.70-4.70	mg/dL
Na	130	136-145	mmol/L
K	3.56	3.5-5.1	mmol/L
Cl	102	98-107	mmol/L
LDH	2635	0-305	U/L
CRP	13.47	<0.33	mg/dL
Venous blood gas analysis			
pH	7.28	7.320-7.430	N/A
PCO2	32.2	32-48	mmHg
PO2	33.8	25-70	mmHg
HCO3	15.6	22.2-28.3	mmol/L
BE	(-)11.5	(-)2-2	mmol/L
HB	11.6	13.5-17.5	g/dL
Na	127	136-145	mmol/L
K	3.5	3.5-5.1	mmol/L
Cl	99	98-107	mmol/L
Ca	1.080	1.1-1.35	mmol/L
Glu	128	N/A	mg/dL
Lac	1.3	1-1.8	mmol/L

The respiratory viruses multiplex polymerase chain reaction panel was positive for rhinovirus. Intravenous (IV) antibiotics were started as sepsis could not be ruled out. The patient was subsequently transferred to a tertiary centre with a presumptive diagnosis of HUS. Further investigations showed a normal coagulation profile, ADAMTS13 activity, homocysteine levels, complement components, and negative anti-factor H antibodies. Blood, urine, and stool cultures were collected.

Due to refractory anuria and hypertension, continuous veno-venous haemodiafiltration (CVVH) was commenced on the second day of hospitalisation. On the fourth day, with the aetiology still undetermined, the patient developed left-sided haemiparesis. A brain computed tomography (CT) scan was performed and showed no abnormalities. Given the rapid progression of neurological manifestations and concern for a severe thrombotic microangiopathy involving the central nervous system, ECZ therapy was initiated despite normal complement levels. On the following day, the patient experienced four focal seizures, prompting the initiation of anticonvulsant therapy and two consecutive PLEX sessions, each followed by ECZ administration.

Also on day 5, stool culture results identified an EPEC positive for the *eae *gene but negative for Shiga toxin production. Virus testing in stool samples was negative. Blood and urine cultures remained sterile, leading to antibiotic discontinuation. A new viral testing was not performed as there was no clinical suspicion of ongoing or progressive viral disease and results would not have altered management. The aetiological investigation is summarised in Tables 2-3.

**Table 2:** Aetiological investigation PT: prothrombin time; APTT: activated partial thromboplastin time; C3: complement component 3; C4: complement component 4; CH50: total haemolytic complement; EPEC: enteropathogenic *Escherichia coli*; N/A: not applicable

Test	Result	Normal range	Unit
PT	11	10.6-11.4	Seconds
APTT	27.7	24-36	Seconds
Fibrinogen	2.2	1.70-4.05	g/L
Homocysteine	6.60	<10	µmol/L
ADAMTS13 activity	0.75	≥0.67	IU/mL
C3	0.91	0.9-1.80	g/L
C4	0.16	0.10-0.40	g/L
CH50	55	>42	U/Ml
Anti-factor H antibodies	0.24	<27	UA/mL
Urine culture	Negative	N/A	N/A
Blood culture	Negative	N/A	N/A
Stool culture	EPEC positive	N/A	N/A
*E. coli* 0157 in stool	Negative	N/A	N/A
*Yersinia enterocolitica* in stool	Negative	N/A	N/A

**Table 3:** Identification of pathogenicity factors and toxins of E. coli by real-time PCR method PCR: polymerase chain reaction; EPEC: enteropathogenic *Escherichia coli*; N/A: not applicable

Pathotype	EPEC
Pathogenicity factors	
eae gene	Positive
aggR	Negative
aatA	Negative
ipaH	Negative
Verotoxins	
vtx1 gene	Negative
vtx2 gene	Negative

**Figure 1:**
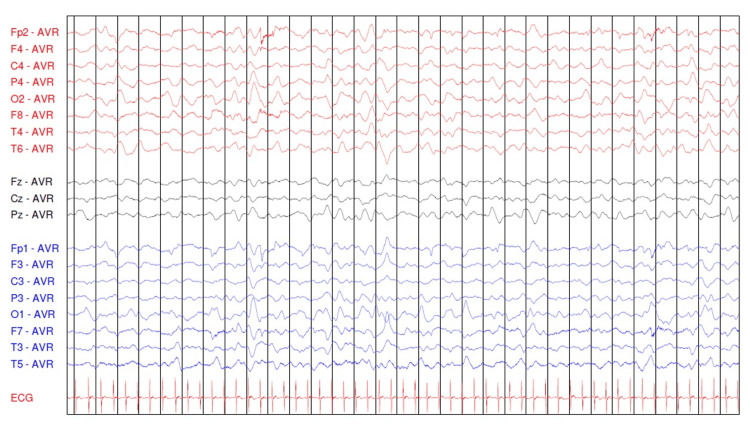
Electroencephalogram in a lethargic child with open eyes

**Figure 2:**
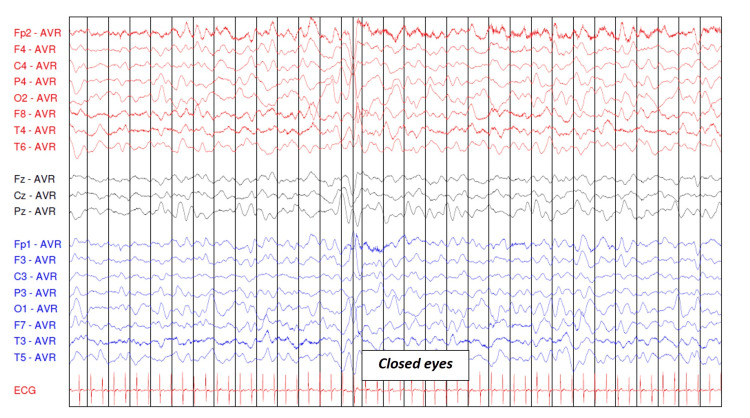
Electroencephalogram in a lethargic child with eyes open and then closed

**Figure 3:**
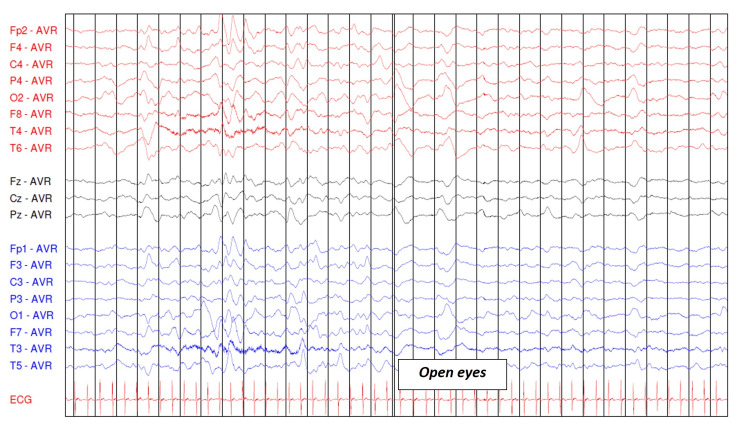
Electroencephalogram in a lethargic child with eyes closed and then open

Electroencephalography (EEG) performed on day 8 revealed diffusely slowed background activity, globally symmetrical and moderately reactive, reflecting diffuse cerebral distress, without epileptiform discharges (Figures 1-3).

Over the subsequent days, the patient demonstrated progressive clinical improvement, with recovery of haematological, renal, and neurological function. He was discharged on day 18, clinically stable and with improved laboratory parameters (Table 4).

**Table 4:** Discharge laboratory evaluation RBC: red blood cells; HB: haemoglobin; HTC: haematocrit; MCV: mean corpuscular volume; MCH: mean corpuscular haemoglobin; MCHC: mean corpuscular haemoglobin concentration; RDW: red cell distribution width; WBC: white blood cells; Na: sodium; K: potassium; Cl: chloride; LDH: lactate dehydrogenase; CRP: C-reactive protein; N/A: not applicable Note that laboratory analyses at admission and at discharge were performed at different institutions, resulting in the use of different measurement units and reference ranges.

Test	Result	Normal range	Unit
RBC	3.33	3.9-5.3	10^12^/L
HB	9.7	11.5-13.5	×10 g/L
HTC	29.8	34-40	%
MCV	89.5	75-87	fL
MCH	29.1	24-30	Pg
MCHC	32.6	31-37	×10 g/L
RDW	17.6	11.5-15.5	%
WBC	10.65	5-15	109/L
Neutrophils	42.5	40-75	%
Lymphocytes	45.9	15-45	%
Monocytes	8.9	2-11	%
Eosinophils	1.9	0-6	%
Basophils	0.8	0-1	%
Platelets	590	200-450	×10^9^/L
Creatinine	0.52	0.3-0.5	mg/dL
Urea	45	11-36	mg/dL
Na	136	136-145	mEq/L
K	4.5	3.40-4.70	mEq/L
Cl	104	98-107	mEq/L
LDH	666	0-305	U/L
CRP	1.4	<5	mg/L

At discharge, he was prescribed ECZ every two weeks, prophylactic amoxicillin, levetiracetam, phenytoin, and enalapril.

At follow-up, the EEG performed one month after discharge was normal, and brain magnetic resonance imaging (MRI) at three months showed only non-specific findings suggestive of chronic microvascular changes. Two months after discharge, he had fully recovered from his motor deficits. From haematological and nephrological standpoints, he remained stable, without the recurrence of HUS, and antihypertensive therapy was discontinued six months after the acute episode.

Eight months after admission, he continued to receive ECZ every two weeks and levetiracetam. Genetic analysis revealed a heterozygous variant of uncertain significance in the DGKE gene and a heterozygous CFHR3-CFHR1 deletion, neither of which was considered causative of the clinical presentation. Therefore, a progressive extension of ECZ dosing intervals was planned.

Nine months after the event, the patient is receiving ECZ every three weeks, with no relapses to date.

## Discussion

This clinical case highlights the diagnostic and therapeutic challenges of HUS when the aetiology remains unestablished and the patient presents in a critical condition, particularly in the presence of severe neurological involvement.

Historically, treatment for CM-HUS relied largely on PLEX, despite the absence of controlled trials demonstrating clear efficacy. Early initiation of PLEX was recommended by the European Paediatric Study Group within 24 hours of diagnosis, and sustained intensive therapy over the first month was associated with partial or complete recovery of renal and haematological parameters in many children. However, a significant proportion still progressed to stage 5 chronic kidney disease. The introduction of ECZ, a humanised monoclonal antibody targeting complement component C5, has revolutionised CM-HUS treatment, with its efficacy first reported in 2009 and later supported by case reports and clinical trials [6].

ECZ is currently approved by the European Medicines Agency and the United States Food and Drug Administration as a standard of care for CM-HUS, as well as for other rare complement-mediated disorders such as paroxysmal nocturnal haemoglobinuria, neuromyelitis optica spectrum disorder, and refractory myasthenia gravis [8].

Current guidelines recommend initiating ECZ within 24-48 hours of disease onset in suspected CM-HUS. If unavailable, PLEX should be started promptly. Importantly, genetic confirmation of complement pathogenic variants is not required prior to initiating ECZ, as the drug is effective even in patients without identifiable complement variants [6].

In contrast, the role of ECZ in IA-HUS remains controversial. To date, no robust randomised clinical trials have consistently demonstrated a clear benefit in STEC-HUS, and ECZ use has not been universally adopted as first-line therapy in this context. Nevertheless, emerging data warrant consideration. The French ECULISHU phase III trial showed that children with STEC-HUS treated with ECZ had a significantly lower incidence of long-term renal sequelae at one year compared to placebo (43.48% vs. 64.44%; p=0.04), suggesting a potential role in reducing chronic kidney damage [5]. Similarly, a study by the Pediatric Nephrology Research Consortium found that among off-label indications for ECZ, only STEC-HUS was associated with a significant improvement in estimated glomerular filtration rate [8]. In addition, numerous case reports describe favourable outcomes in severe presentations, particularly those with neurological involvement, as observed in the present case, where ECZ appeared to support neurological recovery and reduce seizure recurrence [7].

Further support for the potential role of complement inhibition in IA-HUS comes from *Streptococcus pneumoniae*-associated HUS (Sp-HUS). Sp-HUS patients demonstrate dysregulated complement activity secondary to neuraminidase-mediated exposure of Thomsen-Friedenreich antigens, and several case reports and small series have reported good outcomes with this drug in severe or refractory cases [9,10]. Although controlled data are lacking, these observations suggest that ECZ may be beneficial across a broader spectrum of infection-triggered HUS phenotypes, beyond classical CM-HUS.

ECZ is associated with a substantially increased risk of meningococcal infection, with an estimated 1000- to 2000-fold increased incidence. Vaccination is therefore required before treatment initiation, although breakthrough infections remain possible. International consensus guidelines recommend both vaccination and antibiotic prophylaxis for all patients receiving ECZ [6]. In the present case, no additional meningococcal vaccination was administered, as the patient's immunisations were up to date in accordance with the Portuguese National Vaccination Programme, which includes vaccination against meningococcal serogroup B, and were supplemented with vaccination against meningococcal serogroups ACWY. However, prophylactic antibiotic therapy with amoxicillin was initiated following the discontinuation of empirical antibiotic treatment.

Decisions regarding the continuation or discontinuation of ECZ should be guided by the risk of relapse, clinical evolution, renal recovery, patient age, presence of extra-renal involvement, and patient preference. Discontinuation has been shown to be safe after approximately six months of therapy in some patients. While pathogenic variants in complement genes, particularly CFH and MCP, are associated with an increased risk of relapse, they do not appear to influence the initial therapeutic response. Genetic testing, therefore, plays a key role in determining treatment duration, although it should not delay the initiation of ECZ [11].

In the present case, a previously healthy child developed severe HUS with prominent neurological complications triggered by an EPEC strain positive for the *eae* gene but lacking Shiga toxin production. EPEC typically causes diarrhoea by inducing attaching-and-effacing lesions via the intimin protein [12]; however, its association with HUS in paediatric patients is unprecedented in the literature.

It is plausible that, despite the absence of Shiga toxin, this EPEC strain activated alternative inflammatory and coagulation pathways, leading to endothelial injury, complement activation, and thrombotic microangiopathy. The concurrent detection of rhinovirus raises the possibility of an unidentified synergistic interaction between viral infection and EPEC virulence factors, potentially exacerbating vascular and complement-mediated damage.

Genetic testing revealed a heterozygous variant of uncertain significance in the DGKE gene, typically associated with autosomal recessive CM-HUS presenting in infancy, and a heterozygous CFHR3-CFHR1 deletion, which is generally considered a benign finding. Neither variant alone adequately explains the severity of the presentation, supporting the hypothesis of a combined effect of infection-triggered complement dysregulation in a host without a clearly identified genetic predisposition. Nonetheless, it remains plausible that an underlying genetic susceptibility may still exist, due to either pathogenic variants in genes not yet recognised as causal or limitations in current genetic testing methodologies.

From a therapeutic perspective, PLEX was initiated urgently in response to refractory anuria and neurological deterioration, followed by ECZ administration. Although the patient's rapid and complete recovery is encouraging, it remains challenging to attribute clinical improvement exclusively to either modality. This intersection of rarity, clinical severity, and diagnostic uncertainty underscores the complexity of therapeutic decision-making in IA-HUS, particularly regarding the timing and appropriateness of ECZ when robust evidence is lacking.

This case broadens the spectrum of infectious triggers associated with HUS and highlights the importance of a personalised approach to treatment, especially in cases complicated by neurological involvement. Further prospective studies and randomised trials are urgently needed to clarify the role of complement inhibition in non-complement-mediated HUS and to establish evidence-based treatment strategies.

## Conclusions

This case reinforces the importance of a personalised and dynamic approach to the management of HUS, particularly in severe presentations where the underlying cause remains unclear. The association between non-Shiga toxin-producing EPEC and marked neurological involvement is exceptionally rare, emphasising the need for meticulous diagnostic investigation and close clinical monitoring.

Although high-quality clinical trial data remain limited, especially regarding the use of ECZ in IA-HUS, growing evidence from case reports and small studies suggests a potential therapeutic benefit. This likely reflects the central role of complement activation in the pathophysiology of HUS triggered by infectious agents, including in the absence of classic complement gene mutations.

The favourable outcome observed in this child following a combined approach of PLEX and complement inhibition highlights the potential value of early ECZ use in selected cases of HUS with severe extra-renal involvement, particularly when neurological compromise is present. However, the relative contribution of each therapy remains uncertain, and definitive conclusions should await the results of well-designed prospective studies.

Until such evidence becomes available, clinical judgement remains essential in determining the appropriateness of complement-targeted therapy in IA-HUS. Future research should focus on identifying biomarkers that can more precisely predict who may benefit from complement inhibition, thereby guiding personalised treatment strategies in these challenging clinical scenarios.
